# Bone regeneration after transcrestal sinus lift using nanoscale super-hydrophilic versus standard implant surfaces: a single-center, 12-month, parallel controlled clinical trial

**DOI:** 10.1007/s10006-026-01627-4

**Published:** 2026-08-26

**Authors:** Luigi Canullo, Domenico Baldi, Paolo Pesce, Vito Carlo Alberto Caponio, Alessio Triestino, Maria Menini

**Affiliations:** 1 University saint camillus, Rome, Italy; 2 Department of Surgical Sciences (DISC), University of Genoa, Largo Rosanna Benzi, Genova, GE 16100 Italy; 3 Department of Life Sciences, Health and Health Professions, Link Campus University, Rome, Italy; 4 Private practice Rome, Rome, Italy

## Abstract

**Objectives:**

Wettability of dental implants surface might be an additional key variable in the regeneration process and osseointegration. The aim of the study was to evaluate the effect of a new nanoscale roughness, combined with super-hydrophilic surface on bone volume change after trans-crestal sinus floor lift procedure for implant positioning.

**Materials and methods:**

The study was reported according to SPIRIT guidelines. A 12-month, single-center, parallel group clinical trial was conducted to compare changes in bone regeneration and crestal bone level as shown on x-ray, following trans-crestal sinus floor lift using a collagenated hydroxyapatite graft and insertion of single dental implants with two different surfaces: moderately rough surface implants (Control Group – CG) and nanoscale super-hydrophilic surface implants (Test Group – TG). Radiographic bone volume and maximum height of regenerated bone as well as peri-implant crestal bone level were evaluated immediately following implant insertion and after 12 months. A multivariate regression model was built to address the impact of surface characteristics and clinical variables on the outcome.

**Results:**

The study was completed by 25 participants (12 in the TG and 13 in the CG). CG achieved a volume change of 94.54mm^3^ (SD 70.24), while the TG increased by 207.41mm^3^ (SD 168.10) Focusing on bone height gain, CG presented 6.25 mm (SD 1.88), compared to 5.02 mm (SD 1.71) in the TG. For both outcomes, no statistically significant differences were found between the two groups (*p* = 0.087 for bone volume change; *p* = 0.123 for bone height gain). Multivariate linear regression models only partially explained observed variance (respectively 8.6 and 35.4%), resulting in non-statistically significant predictions.

**Discussion:**

Despite the trend of a higher bone volume observed in the hydrophilic surface group, taking into consideration all the variables, this study did not reveal any significant differences in terms of bone regeneration between groups.

**Conclusion:**

Within the limitations of this study, implants with a nanoscale super-hydrophilic surface did not result in significantly greater bone regeneration than conventional moderately rough implants following transcrestal sinus floor elevation, although a trend toward increased bone volume was observed. Further clinical studies are required to clarify the potential regenerative advantages of hydrophilic implant surfaces.

## **Introduction**

Bone augmentation becomes necessary during implant rehabilitation when there is insufficient vertical bone volume. There are essentially two surgical techniques for maxillary sinus elevation: the lateral and the transcrestal approach. The most frequently used and documented technique for the elevation of the sinus maxillary floor is the elevation by the side window, as proposed by Tatum. In 1994, Summers proposed a technique that allowed the elevation of the sinus floor from a crestal access using an instrument called an osteotome, as well as the placement of the implant in the same surgical act [1]. The lateral approach has proven to be effective, with predictable results and high implant survival rates, but it has drawbacks related to the need to create a large flap and in addition carrying out a lateral osteotomy may heighten the risk of complications and prolong postoperative morbidity. The transcrestal approach proves to be advantageous, especially in reducing complications and it is indicated in specific clinical scenarios [2, 3].

A recent systematic review with meta-analysis on patients’ reported outcomes, suggested the crestal approach as the less invasive from patient standpoint [4]. Despite being well-established procedure, it may present possible complications mostly related to the perforation of the sinus membrane [5]. The maxillary sinus is lined by the Schneiderian membrane, which consists of a pseudostratified ciliated respiratory epithelium and acts as a physical barrier and a mechanical clearing system for exogenous agents and particles [6]. Its integrity plays a fundamental role in sinus elevation procedures. Other potential complications, include post-operative infection, sinusitis, graft exposure, graft loss, edema (swelling), seroma formation (accumulation of fluid), bleeding, and membrane exposure [7].

This technique, obviously, requires a proper graft material to maintain the sinus membrane elevated. Recently a new collagenated hydroxyapatite was launched on the market and a recent study presented the reliability of this putty material in the crestal approach [8]. Literature agrees on the fact that implants could be loaded between 3 and 6 months after implant insertion [9, 10]. However, the loading time is strictly correlated to the vertical height of the bony crest, the regenerative potentiality of the sinus (correlated to the medio-buccal sinus width) and the implant surface [2, 11, 12]. The implant surface directly affects the biological interactions with the host and might favor osseointegration. Implant surfaces with higher hydrophilicity and surface energy, qualities that improve cellular adhesion, protein adsorption, and tissue integration at the bone-implant interface, are sometimes referred to be bioactive. Through these physicochemical changes, titanium is transformed from a somewhat bioinert substance into a physiologically active surface that can encourage osseointegration and more advantageous cellular responses [13]. Among these physicochemical changes, hydrophilicity can affect biological processes related to the speed and quality of bone integration. Super-hydrophilic surfaces with a water contact angle of 0° to 10° are typically produced using implant manufacturing processes, however this angle tends to grow over time [14]. Recent evidence support that bioactive surfaces, including hydrophilic ones, could enhance the quality and speed up the osteointegration process. While this enhanced turnover might lead to controversial outcomes in healed sites, it might represent a plus in case of qualitatively and quantitatively compromised bone [15, 16].

Bioactive surfaces may produce, in the case of an augmented site, an increased fibrin network apposition, inducing a substantial accelerated migration and differentiation of osteogenic cells followed by an enhancement of the mineral matrix formation; this could show a tendency toward increased bone turn-over and may lead to a substantial bone regeneration. In vitro and animal studies found that hydrophilic surfaces are advantageous in terms of cell interactions not only for the ability to attract more osteogenic cells [17–19] but also due to macrophages modulation to reduce pro-inflammatory and increase anti-inflammatory gene expression and cytokine production [20]. At the same time, bioactive surfaces could minimize the bacterial adhesion [21]. Despite the promising results from in-vitro and animal models, clinical studies showed inconsistent results [13, 16], and very few studies with controversial results analyzed the indirect effect of a hydrophilic surface on graft integration [22]. A recent study found that a super-hydrophilic implant may lead to a difference in terms of implant survival (even if not significant) and stability of the regenerated bone [23].

In this scenario, the aim of the present study was to assess whether the employment of a super-hydrophilic implant surface in a regenerated sinus, compared to a traditional moderately rough implant, could increase the volume and height of regenerated bone.

## Materials and methods

### Study design

This study was reported in accordance with the SPIRIT [24] (Standard Protocol Items: Recommendations for Interventional Trials) guidelines.

A 12-month, single-center, parallel-arm controlled clinical trial with a 1:1 allocation ratio was undertaken to quantitatively assess bone regeneration following implant placement via the crestal sinus augmentation approach.

### Inclusion/exclusion criteria

Patients requiring single implant insertion in the posterior maxilla in need of sinus elevation were eligible to be included in the study whether they were aged between 30 and 80 years old, either ASA 1 and ASA 2 with a D3 or D4 bone density with a type 1 or 2 post-extraction healed site. Patients had to present healthy periodontal condition (treated periodontitis, PI < 25%, BoP < 25%). The patients were included only if willing to sign an informed consent and participate in the clinical study.

Exclusion criteria included patients requiring immediate post-extraction implant treatment, with uncontrolled periodontitis and treatment delivered to sites where an implant was already failed; patients with self-reported allergy to one or more medicaments to be used during the treatment; pregnancy (confirmed by verbal inquiry); severe smokers (less than 10 cigarettes smoked per day was not considered an exclusion criterion).

The study protocol received approval from the Lazio 1 Ethics Committee (Protocol No. 887/CE Lazio – 25 March 2022) and was registered on ClinicalTrials.gov under the identifier NCT05500911. This manuscript presents a follow-up analysis of the previous published results from the same registered trial, reporting the pre-planned 12-month radiographic secondary outcomes, while the primary endpoint of implant stability has been addressed separately [25].

Written informed consent was obtained from all participants prior to surgical intervention. The study was conducted in accordance with the ethical principles of the Declaration of Helsinki (1975), as amended in 2013.

### Sample size calculation

At the time the study was designed and the sample size calculation was performed, no published clinical studies had specifically investigated the effect of a super-hydrophilic implant surface on bone regeneration following transcrestal sinus floor elevation. Therefore, a pilot study including three patients per group was conducted to estimate the required sample size. To estimate the sample size with an alpha value of 0.05 and power of 80%, means and standard deviations derived from this pilot comparison were input into STATA software (Stata Statistical Software: Release 14. College Station, TX: StataCorp LLC). Command *“power twomeans”* resulted in 6 patients per group (means and SDs, 29.75 ± 33.95 and 320.2 ± 206.03 respectively for the MultiNeo CS (control group, CG) and Multineo NH group (test group, TG). The test implant was characterized by a bioactivated nanoscale super-hydrophilic titanium surface obtained through a proprietary salts and dry technology designed to preserve high surface wettability by minimizing atmospheric contamination during storage. Previous physicochemical characterization demonstrated a homogeneous micro/nanotopography by scanning electron microscopy together with markedly enhanced wettability assessed by contact-angle measurements. These physicochemical properties have been reported elsewhere and were not re-evaluated in the present clinical investigation [26].

The present investigation represents the planned 12-month follow-up of a previously published prospective clinical study evaluating implant stability (ISQ) during the early healing period (baseline, 4 and 6 months). While the previous report focused on osseointegration dynamics, the present study addressed a distinct research question by investigating the volumetric and vertical changes of regenerated bone after one year of healing using CBCT analysis [25]. Out of the 35 patients originally recruited, only 25 accepted to adhere to the second part of the study herein reported, which required the execution of a CBCT at 12-month follow-up to evaluate the volume and height of bone regeneration. Four patients dropped out in the CG group, while 6 patients in the TG group. A total of 25 patients completed the follow-up and were included in the final analysis (against 6 patients per group from sample size estimates). As part of the original study protocol, ISQ values were recorded immediately after implant placement in the bucco-lingual (ISQ_BL) and mesio-distal (ISQ_MD) directions using resonance frequency analysis, together with insertion torque. These measurements have been previously described in detail in the original publication [26]. In the present study, they were not considered study outcomes but were included as baseline covariates in the correlation and multivariable analyses exploring predictors of 12-month bone regeneration.

## Surgical procedure

**Fig. 1:**
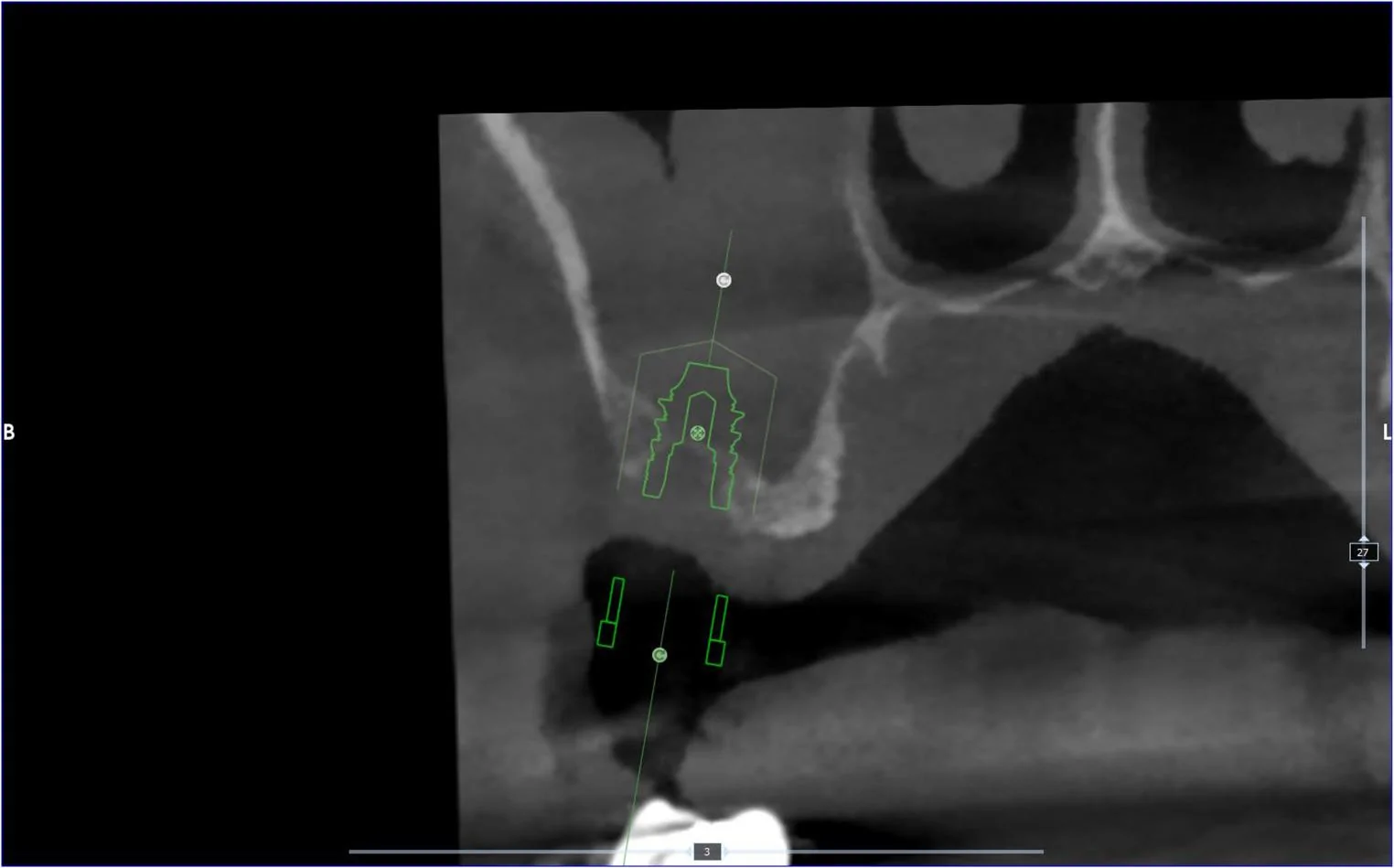
Guided implant surgery project

As described previously, 25 patients underwent CBCT analysis and computer aided planning (Fig. 1). After local anesthesia and flap elevation, sinus floor was reached using calibrated burs. Elevators were utilized to carefully lift the Schneiderian membrane in a gradual manner. Increments of OSSIX™ Bone graft material (5 × 5 × 10 mm), were placed into the created space, moistened with the patient’s blood, and compacted using osteotomes to facilitate additional elevation of the membrane. The integrity of the membrane was preserved and verified through a dental microscope, ensuring controlled pressure for elevation while preventing graft material displacement into the sinus.

Two groups were defined: TG, which received implants with a nanoscale roughness, super-hydrophilic surface (MultiNeO NH CS, Alpha-Bio Tec, Israel), and the CG, which received implants with a conventional moderately rough surface (MultiNeO CS, Alpha-Bio Tec, Israel). Both implant types were manufactured by the same company (Alpha-Bio Tec) and were identical in all aspects except for their surface characteristics. Implant placement was performed using a fully guided osteotomy technique. During the planning phase, residual bone height (in mm) and bone density (in Hounsfield Units) were recorded for each implant using dedicated software (RealGUIDE 5.0, 3Diemme, Cantù, Italy).

Moreover, for every site, the sinus lift was classified as narrow or wide, depending on the extent of the bucco-palatal width at 10 mm from the bone crest [2]. A two-stage surgical approach was followed for submerged healing of the implants. After 6 months the implants were loaded with single crowns.

The patients included in this study underwent same surgical protocol, that was applied to both groups by a single experienced clinician (L.C.) in a private dental clinic in Rome, Italy, between April 2022 and October 2023. Patients were monitored for up to 12 months as part of the study. All data were anonymized, and the treatment groups were labelled generically as Group A and Group B. The dataset for statistical analysis was then sent to V.C.A.C., who was blinded to the treatment characteristics.

### Bone graft 3D evaluation

**Fig. 2:**
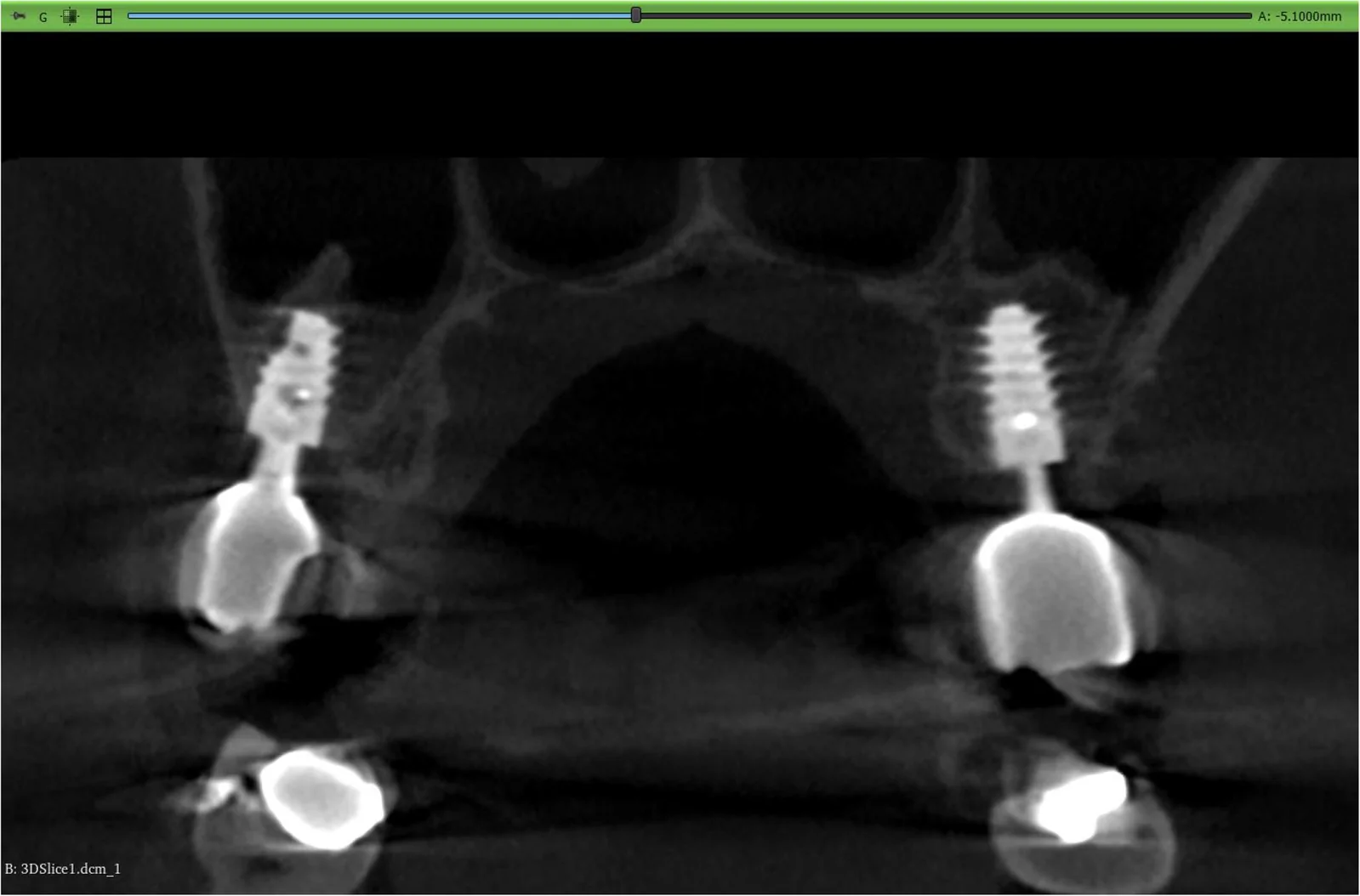
An example of 1-year CBCT evaluation

**Fig. 3:**
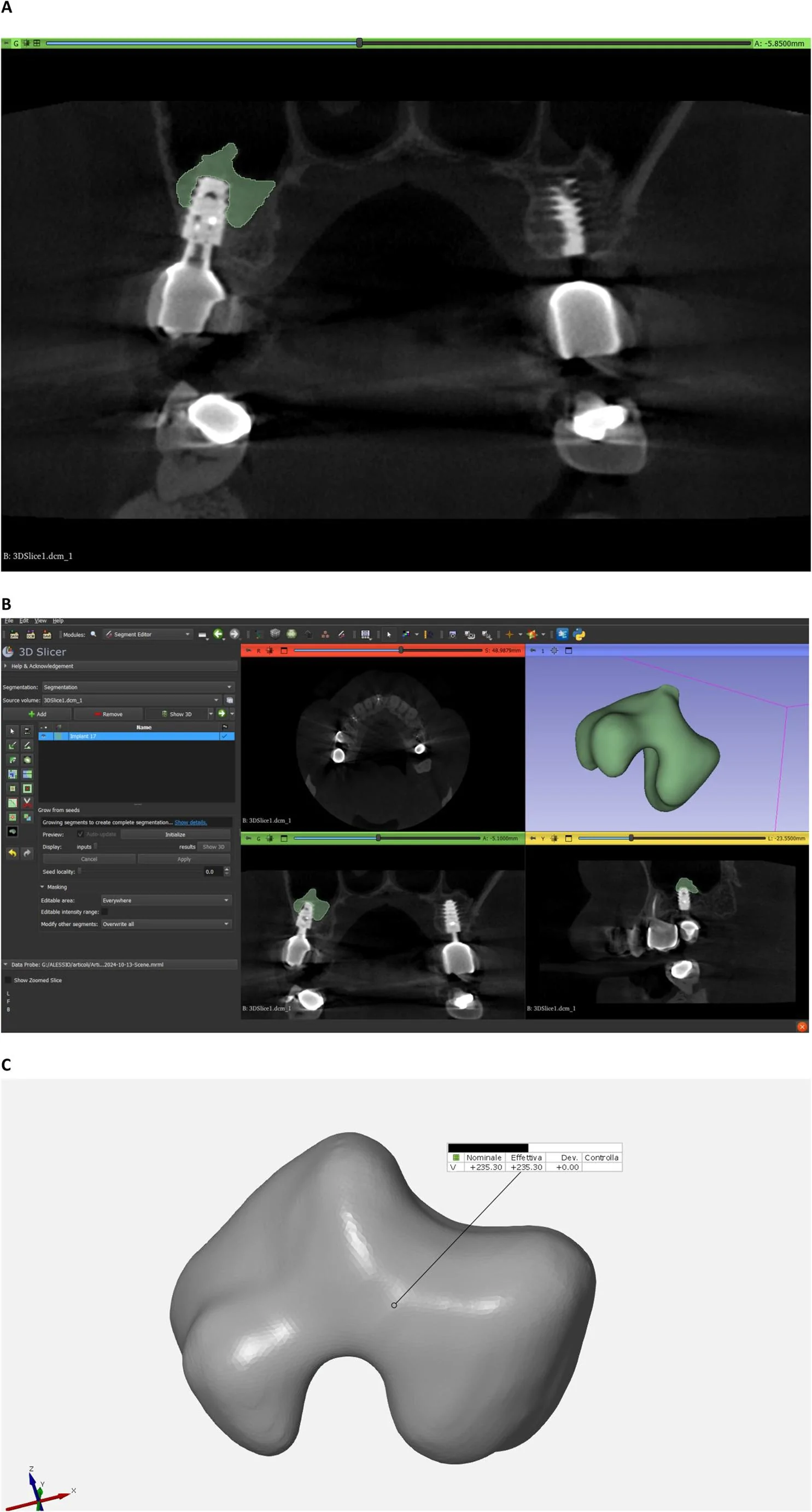
The post operative and pre operative CBCT scans were analyzed. The implant site was analyzed considering the ideal implant axis (as previously calculated through a 3D planning software – Real Guide, 3Diemme) (**A**). After the preliminary phase, the 1-year CBCT was analyzed with a specific segmentation software (3D Slicer, Harvard University). A semi-automatic segmentation technique was performed in order to create a stl file of the regenerated area (**B**) The volume of the regenerated area was calculated with a specific software (GOM Inspect, Zeiss) (**C**)

The formation of new bone was measured by changes in bone height and volume, assessed by CBCT evaluation after 12 months (Fig. 2). For every implant site, the vertical bone height, expressed in mm, was measured. The vertical bone height was measured from the bone crest to the most coronal extent of the bone graft following the implant axis. Tri-dimensional reconstructions of the sinus grafts were performed by semi-automatic segmentation method through an open-source software (3D Slicer, Harvard University, NIH) performed by an experienced operator (A.T.). In order to obtain a reliable reconstruction of the bone graft, a mix of threshold-based segmentation method and manual segmentation method was used. Consequently, for every implant site, a standard triangulation language file (.stl) was created. Graft volume area was evaluated using a specific mesh processing software (Mesh Lab, ISTI-CNR, Italy) and expressed in mm^3^ (Fig. 3a, b, c).

Specifically, the post operative and preoperative CBCT scans were analyzed. The implant site was analyzed considering the ideal implant axis (as previously calculated through a 3D planning software – Real Guide, 3Diemme).

After the preliminary phase, the 1-year CBCT was analyzed with specific segmentation software (3D Slicer, Harvard University). A semi-automatic segmentation technique was performed in order to create a .stl file of the regenerated area.

The volume of the regenerated area was calculated with specific software (GOM Inspect, Zeiss).

### Statistical analysis

Patients were distributed into the two groups (CG and TG). Dichotomous clinical and implant characteristic differences between the two groups were explored by Chi-Square test, while the normal distribution of linear variables was assessed by Shapiro-Wilk test. Results from this test determined the employment of a parametric or non-parametric analysis to test correlation (Spearman or Pearson test) and differences in means (independent-samples T test or Mann-Whitney or Kruskal-Wallis test). To investigate which baseline variables could determine the higher change in 12-months bone volume (dependent variable), a multivariate linear regression model was built. First, we tested univariate linear regression and only those predictors with a p-value < 0.05 were then input in the multivariate model. Similarly, as secondary outcome, change in bone height was investigated.

## Results

A total of 25 patients completed clinical and implant characteristics information at 1-year and were included in the analysis. The TG group was composed of 12 patients while 13 patients belonged to the CG group. Although almost all of patients in the TG group were male, sex difference was not statistically significant (Chi-Square *p* = 0.471). More patients were ASA2 in the CG group (Chi-Square *p* = 0.022). Due to non-normal distribution of linear variables, non-parametric tests were employed. Only insertion torque statistically differed between the two groups (mean CG = 30.6 ± 22.9 vs. TG = 44.4 ± 19, Mann-Whitney *p* = 0.040). All the other variables were not statistically significant between groups and results with either Mann-Whitney test or Chi-Square test p-values are summarized in Table 1.

**Table 1:** Clinical and implant characteristics of patients included in the CG or TG

Variables	Surface						p-value
	CG			TG			
	Mean	Standard Deviation	Median	Mean	Standard Deviation	Median	
Age	58	7	58	61	11	64	0.123
Bone height	5.25	2.21	4.80	5.58	1.38	5.35	0.538
Bone density	373.33	196.99	294.90	484.95	198.36	531.30	0.168
Vestibular diameter	12.86	3.46	13.00	12.08	2.08	12.15	0.611
ISQ_BL	65.92	17.56	72.00	73.08	6.69	73.00	0.406
ISQ_MD	63.00	17.76	64.00	71.58	5.45	71.50	0.225
ISQ_mean	64.46	17.56	67.00	132.71	211.78	72.75	0.205
Implant length	9.15	1.52	8.00	8.83	1.03	8.00	0.769
Implant diameter	4.65	0.56	5.00	4.66	0.52	5.00	0.999
Insertion torque	30.62	22.94	22.00	44.42	19.04	43.00	0.040
Volume change	94.54	70.24	70.82	207.41	168.10	140.53	0.087
Height change	6.25	1.88	6.08	5.02	1.71	4.84	0.123

All the 25 patients adhered to all the follow-up appointments with no further drop-outs.

No implant failed and no biological or technical complications occurred during the 12-month follow-up.

Regarding bone volume change, while CG presented 94.54mm^3^ (SD 70.24), TG revealed 207.41mm^3^ (SD 168.10). Focusing on bone height gain, CG presented 6.25 mm (SD 1.88), TG revealed 5.02 mm (SD 1.71). For both variables, no statistically significant differences were found between the two groups (*p* = 0.087 for bone volume change; *p* = 0.123 for bone height increase).

Correlation coefficients from Spearman Correlation test are described in Table 2. Age was inversely correlated to the vestibular diameter (ρ=-0.526, *p* = 0.007) and the use of narrower implants (ρ=-0.410, *p* = 0.042). Insertion torque correlated to ISQ_BL and ISQ_MD values (respectively, ρ = 0.478, *p* = 0.016 and ρ = 0.443, *p* = 0.026) and inversely to implant length (ρ=-0.425, *p* = 0.034). ISQ_MD correlated to volume change at 12 months (ρ = 0.499, *p* = 0.011). Lastly, bone change at 12 months inversely correlated with bone height at the beginning of the study (ρ=-0.555, *p* = 0.004).

**Table 2:** Spearman rank correlations among the linear variables investigated in this study

		Age	Bone height	Bone density	Medio-buccal diameter	ISQ_ BL	ISQ_ MD	ISQ_ mean	Implant length	Implant diameter	Insertion torque	Volume change	Height change
Age	corr coeff	1.000	0.070	0.295	-0.526^**^	-0.381	-0.233	-0.301	-0.122	-0.410^*^	0.049	0.019	**-0.317**
	Sig.(2-tailed)		0.740	0.153	0.007	0.060	0.262	0.144	0.561	0.042	0.818	0.929	**0.122**
Bone height	corr coeff		1.000	-0.155	-0.208	0.186	0.113	0.184	0.144	-0.353	-0.126	-0.061	**-0.555** ^**^
	Sig. (2-tailed)			0.458	0.318	0.372	0.592	0.378	0.493	0.084	0.549	0.773	**0.004**
Bone density	corr coeff			1.000	-0.143	-0.208	-0.232	-0.212	-0.193	0.359	0.208	-0.068	-0.001
	Sig. (2-tailed)				0.496	0.319	0.265	0.308	0.354	0.078	0.318	0.748	0.997
Medio Buccal diameter	corr coeff				1.000	0.126	0.020	0.086	-0.092	0.138	0.052	0.140	0.194
	Sig. (2-tailed)					0.549	0.925	0.684	0.662	0.509	0.805	0.503	0.352
ISQ_BL	corr coeff					1.000	**0.900** ^**^	**0.972** ^**^	-0.079	0.189	**0.478** ^*^	0.278	-0.147
	Sig. (2-tailed)						**0.000**	**0.000**	0.707	0.364	**0.016**	0.179	0.484
ISQ_MD	corr coeff						1.000	**0.964** ^**^	-0.053	0.106	**0.443** ^*^	**0.499** ^*^	-0.083
	Sig. (2-tailed)							**0.000**	0.803	0.615	**0.026**	**0.011**	0.694
ISQ_mean	corr coeff							1.000	-0.064	0.135	**0.485** ^*^	0.382	-0.135
	Sig. (2-tailed)								0.763	0.521	**0.014**	0.059	0.520
Implant length	corr coeff								1.000	-0.162	**-0.425** ^*^	-0.111	0.339
	Sig. (2-tailed)									0.440	**0.034**	0.596	0.098
Implant diameter	corr coeff									1.000	0.374	0.061	0.379
	Sig. (2-tailed)										0.065	0.773	0.062
Insertion torque	corr coeff										1.000	0.385	-0.053
	Sig. (2-tailed)											0.057	0.803
Volume change	corr coeff											1.000	-0.068
	Sig. (2-tailed)												0.748
Height change	corr coeff												1.000
	Sig. (2-tailed)	1.000	0.070	0.295	**-**,526^**^	-0.381	-0.233	-0.301	-0.122	**-**,410^*^	0.049	0.019	-0.317

*. Correlation is significant at the 0.05 level (2-tailed)

**. Correlation is significant at the 0.01 level (2-tailed)

The multivariate linear regression studied as first outcome the volume change. The model included age of patients, bone height and density, vestibular diameter, ISQ_BL and MD, implant length and diameter, torque and patients’ sex, ASA and treatment group (CG vs. TG). The overall model was characterized by a satisfactory level of prediction (*R* = 0.76) and explained overall the 58.1% of the variance. However, when this was adjusted for the number of variables, this dropped, explaining the 8.6% of variance in volume bone change at 12 months, however not statistically significant nor at total model (*p* = 0.400) nor for single predictors (Table 3). Similarly, for the bone height change prediction, the model achieved a substantial value (*R* = 0.83) and explained the 70.4% of variance, and still 35.4% of adjusted variance without statistical significance (*p* = 0.126). The only predictor that resulted statistically significant was implant length, however, when adjusting for Bonferroni correction, this predictor lost significance (Table 4).

**Table 3:** Coefficients related to the variables included in the multivariate linear model to predict volume change at 12 months follow-up

Model	Coefficients^a^						
	Unstandardized Coefficients		Standardized Coefficients	t	Sig.	95,0% Confidence Interval for B	
	B	Std. Error	Beta			Lower Bound	Upper Bound
(Constant)	963.506	874.095		1.102	0.294	-960.364	2887.377
Age	-0.879	4.729	-0.057	-0.186	0.856	-11.287	9.529
Bone height	0.960	17.021	0.013	0.056	0.956	-36.503	38.422
Bone density	-0.057	0.170	-0.085	-0.339	0.741	-0.431	0.316
Medio-buccal diameter	3.147	12.611	0.066	0.250	0.808	-24.609	30.904
ISQ_BL	-4.500	11.836	-0.451	-0.380	0.711	-30.551	21.551
ISQ_MD	6.586	11.618	0.664	0.567	0.582	-18.985	32.157
ISQ_mean	-0.120	0.264	-0.130	-0.456	0.657	-0.700	0.460
Implant length	-21.831	25.111	-0.206	-0.869	0.403	-77.099	33.437
Implant diameter	-137.447	103.093	-0.532	-1.333	0.209	-364.353	89.459
Insertion torque	-0.317	2.097	-0.051	-0.151	0.882	-4.933	4.298
Dummy sex	-170.258	93.008	-0.592	-1.831	0.094	-374.968	34.452
Dummy treatment (CG vs. TG)	55.780	72.479	0.208	0.770	0.458	-103.745	215.306
Dummy ASA	-62.439	69.875	-0.228	-0.894	0.391	-216.234	91.355

a. Dependent Variable: volume change

**Table 4:** Coefficients related to the variables included in the multivariate linear model to predict bone height change at 12 months follow-up

Model	Coefficients^a^						
	Unstandardized Coefficients		Standardized Coefficients	t	Sig.	95,0% Confidence Interval for B	
	B	Std. Error	Beta			Lower Bound	Upper Bound
(Constant)	-10.074	10.028		-1.005	0.337	-32.146	11.999
Age	-0.002	0.054	-0.010	-0.037	0.971	-0.121	0.117
Bone height	-0.316	0.195	-0.309	-1.618	0.134	-0.746	0.114
Bone density	0.001	0.002	0.087	0.414	0.687	-0.003	0.005
Medio-buccal diameter	0.106	0.145	0.161	0.732	0.480	-0.213	0.424
ISQ_BL	-0.237	0.136	-1.736	-1.742	0.109	-0.535	0.062
ISQ_MD	0.251	0.133	1.855	1.884	0.086	-0.042	0.544
ISQ_mean	0.000	0.003	0.009	0.036	0.972	-0.007	0.007
Implant length	0.797	0.288	0.551	2.766	0.018	0.163	1.431
Implant diameter	1.858	1.183	0.527	1.571	0.144	-0.745	4.461
Insertion torque	-0.012	0.024	-0.138	-0.489	0.634	-0.065	0.041
Dummy sex	1.383	1.067	0.352	1.296	0.222	-0.966	3.732
Dummy treatment (CG vs. TG)	-0.937	0.832	-0.256	-1.126	0.284	-2.767	0.894
Dummy ASA	0.148	0.802	0.039	0.184	0.857	-1.617	1.912

a. Dependent Variable: height change

## Discussion

In this 12-month, single center, parallel group clinical trial there were no statistically significant differences in the bone regeneration following the insertion of an implant through a crestal sinus lift approach. At the end of the study, the bone volume gain in CG was 94.54mm^3^ (SD 70.24), while TG revealed 207.41mm^3^ (SD 168.10) and the height gain in the CG presented 6.25 mm (SD 1.88), while TG revealed 5.02 mm (SD 1.71), so that in case of transcrestal sinus lift, a hydrophilic surfaced implant failed to present any additional advantage compared to traditional moderately roughly surfaced implant.

The present investigation extends the findings of our previous 6-month report by evaluating the stability of regenerated bone after one year of healing. While the previous study focused on implant stability during the early osseointegration phase, the present analysis assessed the maintenance and maturation of the regenerated bone following functional loading. Bone remodeling after sinus augmentation is a dynamic process that continues beyond the initial healing period, making a 12-month evaluation clinically relevant. The present results confirmed the absence of statistically significant differences between implant surfaces that had already been observed during early healing. Therefore, the longer follow-up reinforces the conclusion that, although nanoscale super-hydrophilic implants showed a consistent numerical trend toward greater bone regeneration, this advantage did not reach statistical significance within the first year.

The present findings corroborate those of Qian et al., despite slight methodological differences between the two studies. Although our findings are consistent with those reported by Qian et al. [23], the two studies differ substantially in design and clinical setting. Qian et al. conducted a retrospective study evaluating hydrophilic implants placed simultaneously with lateral sinus floor elevation and reported clinical and radiographic outcomes over a follow-up ranging from 2 to 5 years. In contrast, the present investigation was designed as a prospective controlled clinical study focusing on transcrestal sinus floor elevation, a less invasive technique characterized by different biological and surgical features. Moreover, while the previous study primarily assessed long-term clinical and radiographic outcomes, the present work specifically investigated volumetric bone regeneration and vertical bone gain during the first year of healing using standardized CBCT-based three-dimensional analysis. Therefore, although both studies suggest a numerical advantage of hydrophilic implant surfaces without reaching statistical significance, the present investigation provides complementary evidence in a different surgical scenario and during the critical phase of graft maturation and early functional loading.

All the differences mentioned could be explained by the hydrophilic surfaced implant to accelerate the regenerative cascade. In fact, several studies confirmed the capability of super-hydrophilic titanium implants to recall quantitatively more cells [13]. At the same time, the faster and numerically higher titanium/cell interactions were demonstrated to allow a better tissue layering and, then, a faster integration [27]. However, if the effect of bioactivation of the surface could be easily demonstrated in healed sites, the same cannot be said in case of sites to be regenerated. A recent study on animal model failed to reach any significance in post-extractive sites, although a numerical difference between groups was present [28].

In addition, Qian et al. demonstrated that the “physiologic” shrinkage, following graft remodeling, appeared smaller in the sinuses treated with hydrophilic implants, although not significant [23]. This data was not reported in our study due to ethical reasons, however it could be supposed to show similar behavior also in the present analysis. The graft adopted in the study is OSSIX™ Bone which was demonstrated to present a contraction rate of approximately 20% in normal conditions after 12 months [8]. A hydrophilic surface, could possibly reduce this shrinkage rate enhancing the cell turnover. OSSIX™ Bone is an novel cross-linked collagen sponge designed to support bone formation while preserving bone quality. It provides a natural environment for cellular migration, proliferation, and maturation, promoting optimal bone regeneration. After a 4-month healing period, the newly formed bone exhibits characteristics comparable to natural alveolar bone, as no minimal residual material or dense mineralized graft particles remain [29], in comparison with data in the literature reporting substantial amounts of non-resorbed particles of conventional biomaterials at the end of the healing process [30].

Minimal number of perforations (1 in test and 1 in control group) occurred during sinus membrane elevation. However, the use of a putty material in both groups allowed to prevent any complication described in the literature, and seemed not to interfere with the regenerative processes [31, 32].

Although the study fulfilled the estimated sample size requirement, the larger-than-expected variability observed in both groups reduced the precision of the estimates. Therefore, while the present findings suggest no statistically significant differences between the investigated implant surfaces, modest treatment effects cannot be completely excluded. In the present prospective controlled study, the sample size was smaller than the retrospective one in Qian et al. However, all the variables involved in sinus bone regeneration (age, patient health condition, medio-buccal width, perforation of the Schneiderian mucosa, graft material) were preoperatively analysed to create two homogeneous groups. This may allow us to consider reliable the reported outcomes, then balancing the paucity of the sample size. Additionally, the high standard deviation in both groups may jeopardize any possibility to reach the significance. Indeed, the multivariate linear regression model only explained partially the observed variance, for which underexplored variables may further contribute to the outcomes (respectively 8.6 and 35.4% for total volume and bone height change).

However, the main limitation of the present study was represented mostly by the outcome measure analysis. Although CBCT provided precise volumetric assessment, this measurement seems to appear only speculative [33]. In fact, 3D volumetric radiography fails to clarify the quality of bone regeneration. To clearly understand if a hydrophilic surface may influence the endo-sinus integration of a graft material, only a histological analysis might be so sensitive and conclusive. However, in this case, ethical reasons could appear obstructive.

## Conclusions

Within the limitations of this study, implants with a nanoscale super-hydrophilic surface did not result in significantly greater bone regeneration than conventional moderately rough implants following transcrestal sinus floor elevation, although a trend toward increased bone volume was observed. Further studies with a larger number of patients are required to clarify the potential regenerative advantages of hydrophilic implant surfaces.

## Data Availability

Data available on request.
